# Progress in Genito-Urinary Surgery

**Published:** 1904-04-16

**Authors:** 


(*To be continued.*)

### Progress in Genito-Urinary Surgery.

(*Continued from Page* 29.)

**Asepsis in Catheterisation and Cystoscopy.**—Leopold Casper (Berlin)[8] recommends the following methods of sterilising catheters and cystoscopes. Metal catheters to be boiled in water, soft catheters in saturated solution of sulphide of ammonium for five minutes, but the latter must not be preserved in the same solution or they will become rough and too soft. Or soft instruments may be each wrapped up separately in a cloth and placed for two hours in a steam steriliser, and then they may be kept in the cloths until required. The author cleans cystoscopes by Gerson's method, viz., by three rubbings, each of a minute's duration, with cloths soaked in spirits of soap and keeping the instrument wrapped up in such cloths until required for use. As a catheter lubricant a mixture of oxycyanate of mercury (1 in 500) in glycerine, tragacanth, and water is recommended. For prophylactic washing of the healthy bladder, 100 or 200 cc. of a solution of silver nitrate (1 : 1000-2000) is used. For cystoscopy the bladder is washed out with oxycyanate of mercury, 1 in 5000.

**Papilloma of the Bladder.**—R. Frank[9] describes a case in which a new method of removing a papilloma of the bladder was successfully practised. The patient, a man of 63, very anæmic from hæmaturia, had such a tumour at the orifice of the right ureter. The operation consisted in separating the rectum from the prostate, after the method of Zuckerkandl, up to the posterior wall of the bladder. The fold of Douglas was pushed upwards. A longitudinal median incision was then made into the bladder between the vesiculæ seminales, the edges of the incision everted, and the pedicle of the papilloma seized and separated with the Paquelin cautery. A drainage-tube was placed in the wound, with gauze packing around it, and a catheter tied in the bladder. The former were removed in seven days, the catheter in 14 days. Complete healing followed. The author has employed the same method for the removal of vesical calculi and considers it to be less dangerous than supra-pubic cystotomy, especially in old people, and equally good for the purposes required. The operation causes but little bleeding and can be quickly performed. The after treatment is simple, and drainage of the urine is in the direction of gravity.

**Spontaneous Fracture of Vesical Calculi.**—G. Kapsammer[10] states that spontaneous fracture of vesical calculi is very rare. In literature he was only able to find 60 indubitable cases recorded. With two exceptions the stones in these cases were composed of urates. The causes of spontaneous fracture are not known. Kapsammer records a case, that of a man of 74, who suffered from very severe vesical tenesmus. By suprapubic cystotomy 49 fragments of stone were removed which showed smooth external surfaces and flat fracture surfaces, and were composed of uric acid. From the 49 fragments ten complete faceted calculi could be constructed. The smooth fracture surfaces could only, it would seem, have been produced by mechanical force, that of the bladder contractions.

**Perineal Section for Stricture of the Urethra.**—Chas. C. Miller (Chicago),[11] has discussed this procedure. The operation is an easy one if the section can be made upon a staff, and in strictures of small calibre prolonged efforts should be made to enlarge the stricture sufficiently to permit of the passage of a staff. If a filiform guide can be passed, no better method can be found for this than the use of the Maissoneuve urethrotome. Having passed the staff and divided the floor of the stricture, the roof of the latter is to be examined for stricture tissue, and that tissue divided. The Paquelin cautery at a dull-red heat is now carried to the bottom of the wound in the roof of the urethra and one surface of this wound rapidly stroked with the heated blade. Then one margin of the incision in the floor of the urethra is similarly treated. The use of the cautery is to insure the formation of granulations and thus prevent primary union taking place. The object in dividing a stricture is to alter the nutrition of the tissue, so that absorption may occur, or to add to the calibre of the urethra by the production of new tissue developed from granulation tissue. A catheter, not smaller than a No. 22F, is then passed into the bladder through the wound, so that the eye is just in the bladder, and is fixed with a stitch to the skin posteriorly. The remainder of the wound is closed with sutures. The author prefers not to leave the catheter in the bladder after the patient comes round from the anæsthetic, but instead to catheterise the bladder through the perineum every six hours for three days. It is important to have the posterior part of the wound clean cut, not chopped up, otherwise it may be difficult to pass bougies subsequently. The sounds used every few days afterwards should have a short beak and a slight curve.

[8] (Abst.) Munchener Med. Wochenschrift, Nov. 24, 1903, p. 2063. [9] (Abst.) Zentralb. f. Chir., No. 41. Oct. 10, 1903, p. 1138. [10] Ibid. [11] Med. Rec., Nov. 28, 1903, p. 854.

